# Comparison of fish and oil supplements for a better understanding of the role of fat level and other food constituents in determining bioaccessibility

**DOI:** 10.1002/fsn3.894

**Published:** 2019-03-13

**Authors:** Romina Gomes, Sara Martins, Cláudia Afonso, Narcisa M. Bandarra, Carlos Cardoso

**Affiliations:** ^1^ Division of Aquaculture and Upgrading (DivAV) Portuguese Institute for the Sea and Atmosphere (IPMA, IP) Lisbon Portugal; ^2^ Agronomy Superior Institute University of Lisbon Lisbon Portugal; ^3^ CIIMAR Interdisciplinary Centre of Marine and Environmental Research University of Porto Porto Portugal

**Keywords:** bioavailability, fat content, fatty acids, fish, oil supplements

## Abstract

In order to investigate the effects of fat level and protein and other components on lipid bioaccessibility, the bioaccessibility of total lipids and particular fatty acids (FAs) of fish samples with different fat levels (5.4% w/w, 10.2% w/w, and 16.6% w/w) and cod liver oil supplement in different quantities (82, 154, 313, 604, and 1,027 mg) was determined by an in vitro digestion model. Digestion of the fish and oil (up to 154 mg) samples as measured by TAG disappearance was complete. Lipolysis was impaired by high amounts of oil (313 mg and higher). Bioaccessible FA profiles had similarities with the initial (before digestion) FA profiles. However, total MUFA and oleic acid contents were higher in the bioaccessible fraction. The bioaccessibility of EPA and DHA was generally lower than that of oleic acid and total MUFA. Fat level did not affect FAs’ bioaccessibility. On the other hand, protein and other components may have interfered in lipid bioaccessibility and it was found that the reduction of bioaccessibility was stronger when the ratio of the lipid fraction to the nonlipid fraction (mainly protein) was smaller.

## 1 INTRODUCTION

Fish contain significant amounts of ω3 polyunsaturated fatty acids (ω3 PUFAs), namely eicosapentaenoic acid (EPA, 20:5 ω3) and docosahexaenoic acid (DHA, 22:6 ω3), which are associated with decreased morbidity and mortality from cardiovascular and other diseases as well as with fetal development (Simopoulos, 2002). Given this, several public health organizations have recommended adequate levels of consumption for these FAs.

In order to assess the health effects of fish and supplements, it is crucial to attain information concerning the FA concentrations that are effectively absorbed by the human body (Cardoso, Afonso, Lourenço, Costa, & Nunes, 2015). Bioaccessibility can be seen as an indicator for the maximal oral bioavailability—share of a given FA that reaches the systemic circulation and becomes available to the tissues where it is needed, such as the nervous tissue—or as an upper limit of the oral bioavailability for any given food constituent, that is, the fraction of that constituent reaching systemic circulation (Cardoso et al., 2015). Depending on numerous factors such as type and processing of food or presence of certain antinutritional constituents, the studied food constituent, such as a given FA, may be more or less bioavailable (Afonso et al., 2015; Van Het Hof, West, Weststrate, & Hautvast, 2000; Wienk, Marx, & Beynen, 1999). This justifies a greater attention to the issue of FA composition and FA bioaccessibility—the share of a given FA initially present in a food that is rendered available for absorption across the intestinal wall after the human digestive process (Cardoso et al., 2015)—in seafood and processed products obtained from seafood, such as cod liver oil supplements.

Recent experimental work has been undertaken with the aim of finding and improving suitable in vitro digestion models for the realistic simulation of the human digestive system (Cardoso et al., 2015; Minekus et al., 2014). Such challenge is compounded with extra technical difficulties when the targeted food constituent is also present in the substances used in the simulation of digestion, as in the case of lipids, which are to be found, for instance, in the bile (Afonso et al., 2015). For lean seafood, this can pose problems, since the amount of bile lipid components will not be much lower than the quantity of lipids in the sample. To the knowledge of the authors, this issue has not been conclusively covered by the literature. Indeed, these are only a few studies on lipid bioaccessibility (Cardoso et al., 2015; Costa et al., 2015). Besides, lipid class distribution of the fatty acids (FAs) may be influential for the bioaccessibility of particular FAs, thereby putting another layer of complexity onto the issue. For instance, it has been observed in in vivo studies with humans that FAs bound in triacylglycerol (TAG) are more bioavailable than FAs bound in ethyl ester (EE) form (Dyerberg, Madsen, Møller, Aardestrup, & Schmidt, 2010; Neubronner et al., 2011). These EEs can be found in fish oil supplements, but not in the large majority of foods. Finally, the interference of other nutrients, such as proteins, in FA bioaccessibility has been suggested—especially after drastic thermal treatment that can generate protein aggregates—but not decisively proven (Afonso et al., 2015; Costa et al., 2015, 2016).

Therefore, for a deeper understanding of the factors affecting FA bioaccessibility, fish with different levels of fat and cod liver oil supplement at different amounts were chosen as models for highlighting the impact of fat level and of the presence of proteins and other components on digestibility of seafood as assessed by an in vitro model. Raw fish was preferred to cooked fish in order to exclude the effects of thermal treatment from the comparison between fish and oil, thereby highlighting the presence of other components besides lipids as a major independent variable.

## 2 MATERIALS AND METHODS

### 2.1 Experimental design and samples

For assessing the effect of different fat levels on the in vitro human digestion model and FA bioaccessibility, fish samples with three different fat levels were selected for being used as substrate for the digestive simulation. Accordingly, fish samples corresponding to approximately 5, 10, and 15% w/w fat were chosen and termed A, B, and C, respectively. Moreover, a cod liver oil supplement was also used as substrate for the digestion model and different amounts of oil (82, 154, 313, 604, and 1,027 mg) were subjected to the digestive procedure. This enabled to assess the effect of fat on bioaccessibility without the interference of other nutrients, such as proteins, present in the fish samples. On the other hand, the juxtaposition of the three fish samples to three different amounts of supplement enabled to compare situations with a similar fat input in the digestive system—1.5 g of fish, corresponding to fish with 5% w/w—75 mg, 10% w/w—150 mg, and 20% w/w—300 mg of fat, was matched by 82, 154, and 313 mg of cod liver oil supplement—but distinct food chemistry, that is, to evaluate the importance of the other components (protein, minerals, etc.) in the bioaccessibility of FAs. For this reason, raw fish was used instead of cooked fish—otherwise, the effects of thermal treatment would be impossible to disentangle from the fish matrix effects. Regarding the higher tested amounts of oil supplement, 604 mg was chosen for simulating fish with fat content above 20% w/w and 1,027 mg corresponded to the amount of fat in many cod liver oil prescriptions.

### 2.2 Bioaccessibility

For the bioaccessibility study of compounds, raw fish (samples A, B, and C) and cod liver oil (supplement) samples were used. In order to acquire information about FA bioaccessibility, samples were subjected to an optimized in vitro digestion methodology previously described (Afonso et al., 2015). In particular, the sample amount used as input to the model (1.5 g) was chosen on the basis of previous studies aiming at an optimization of the digestive model. These studies showed that 1.5 g corresponded approximately (in average) to a 150 g meal (typical fish portion size) and 3.0 g corresponded to a half empty stomach after eating a warm meal with different components (e.g., fish plus rice and vegetables) and, also, to a caloric content of 400–500 kcal, such as was initially modeled by other authors (Versantvoort, Oomen, Van de Kamp, Rompelberg, & Sips, 2005). In the current study, since the focus was solely on fish vs. oil per se, the 150 g meal situation was chosen. The composition and chemicals used for the simulated digestive juices (saliva, gastric, duodenal, and bile) were the same described by Afonso et al. (2015). Briefly, 1.5 g of fish (a raw, solid piece cut from a fillet) or the selected amount of liquid oil (82, 154, 313, 604, and 1,027 mg) was homogenized with 4 ml of artificial saliva using a model Polytron PT 6100 homogenizer (Kinematica, Luzern, Switzerland) at a velocity of 10,000 rpm during 3 min (mouth phase simulation). Then, 8 ml of artificial gastric juice was added, before proceeding to 2‐hr incubation at 37°C (gastric phase simulation). Afterward, 8 ml of artificial duodenal juice (containing porcine pancreatic lipase, PPL) plus 4 ml of artificial bile and 1.3 ml of NaHCO_3_ was added and followed by a second incubation at 37°C during 2 hr (intestinal phase simulation). The digested and non‐digested fractions were separated by a centrifugation at 2,750 × *g* for 5 min.

### 2.3 Calculations

The percentage (%) of FAs in the bioaccessible fraction was calculated as follows:

$$\%\;\text{FA bioaccessible}={\left[\text{FA}\right]}_{\mathrm{bioaccessible}}\times 100/{\left[\text{FA}\right]}_{\mathrm{initial}}$$

Where:

[FA]_bioaccessible_ – [FA] in the bioaccessible fraction
[FA]_initial_ – [FA] in the fish or oil sample before the digestion.

The amount of FA present in the enzyme solution was taken out from [FA]_bioaccessible_.

### 2.4 Analyses

#### 2.4.1 Moisture, ash, protein, and lipid contents

AOAC methods (AOAC, 2005) were applied to the determination of moisture and ash levels. The protein level was quantified using a combustion method of analysis with the FP‐528 DSP LECO nitrogen analyzer (LECO, St. Joseph, USA) calibrated with EDTA according to the Dumas method (Saint‐Denis & Goupy, 2004). Total fat was determined following the Bligh and Dyer method (Bligh & Dyer, 1959) using methanol and chloroform as solvents.

#### 2.4.2 Lipid extraction

Lipid extraction from fish samples was done by the Bligh and Dyer method (Bligh & Dyer, 1959). For fat extraction in the bioaccessible fraction, a different technique was used, since fat is already available in the bioaccessible phase due to mechanical and enzymatic action during the in vitro digestion. Briefly, 2 ml chloroform was added followed by centrifugation (Sigma 3k30, Osterode am Harz, Germany) at 2,000 × *g* for 5 min at a temperature of 4°C. After repeating previous step, organic phase was filtered through anhydrous sodium sulfate and evaporated in a rotary evaporator (Rotavapor, Büchi RE 121, Flawil, Switzerland). The samples were stored at −20°C until further analyses. Afterward, the fatty acid profile for each main lipid class was determined.

#### 2.4.3 Lipid class determination

The relative weight of each lipid class was determined by analytical thin‐layer chromatography (TLC) using a previously described method (Bandarra, Batista, Nunes, & Empis, 2001). An eluent mixture of hexane: diethyl ether:acetic acid (50:50:2 by volume) and a plate coated with 0.25 mm silica gel G were used. Lipid class identification was done by comparison with standards (Sigma Chemical Co., St. Louis, MO, USA). The relative percentage of each lipid class was determined using a GS‐800 densitometer and version 4.5.2 of Quantity One 1‐D Analysis Software from Bio‐Rad (Hercules, CA, USA).

#### 2.4.4 Fatty acid profile

The fatty acid profile was determined in the samples before and after digestion (bioaccessible fraction). Fatty acid methyl esters (FAME) were prepared by acid‐catalyzed transesterification using the methodology described by Bandarra, Batista, Nunes, Empis, and Christie (1997). Samples were injected into a Varian Star 3800 Cp gas chromatograph (Walnut Creek, CA, USA), equipped with an autosampler with a flame ionization detector at 250°C. FAME were identified by comparing their retention time with those of Sigma‐Aldrich standards (PUFA‐3, Menhaden oil, and PUFA‐1, Marine source from Supelco Analytical). Data in mg/100 g of edible part were calculated using the peak area ratio (% of total fatty acids) and the lipid conversion factors set by a previous study (Weihrauch, Posati, Anderson, & Exler, 1977).

### 2.5 Statistics

All data were analyzed using Statistica 6 software (StatSoft, Inc., Tulsa, OK74104, USA). One‐way ANOVA was used to determine significant differences (*p *<* *0.05) between fish and cod liver oil supplement samples before digestion, and factorial ANOVAs were applied to determine significant differences (*p *<* *0.05) between fatty acids in combination with the effect of digestion as well as between bioaccessibility of different fatty acids in the same sample combined with the bioaccessibility of the same fatty acid among different fish and cod liver oil samples, followed by a multiple comparison test (Tukey HSD). When data could not satisfy normal distribution and homoscedasticity requirements, differences were analyzed with nonparametric analysis of variance (Kruskal–Wallis) followed by nonparametric multiple comparison test (Zar, 1999).

## 3 RESULTS AND DISCUSSION

### 3.1 Characterization of samples

#### 3.1.1 Proximate composition

The proximate composition of the analyzed fish samples (A, B, and C) is shown in Table 1. As expected, the moisture content was highest in the fish A sample and lowest in the fish C sample, thereby corresponding to the opposite of the fat content, which increased from sample A to sample C. The ash content variation displayed the same trend observed for moisture content. The protein concentration was lower only in the fattest fish sample (C).

**Table 1:** Proximate composition (%, w/w) and fatty acid profile (% of total FAs) of the three fish samples with different fat contents and the cod liver oil supplement

	Fish A	Fish B	Fish C	Cod liver oil supplement
Moisture	71.1 ± 0.2^c^	66.3 ± 0.1^b^	64.8 ± 0.0^a^	—
Protein	20.9 ± 0.1^b^	22.9 ± 0.7^b^	17.8 ± 0.7^a^	—
Lipid	5.4 ± 0.3^a^	10.2 ± 0.1^b^	16.6 ± 0.2^c^	—
Ash	1.6 ± 0.0^c^	1.5 ± 0.0^b^	0.8 ± 0.0^a^	—
14:0	7.6 ± 0.4^b^	6.7 ± 1.5^b^	2.1 ± 0.0^a^	6.2 ± 1.2^ab^
16:0	17.3 ± 0.2^c^	20.0 ± 1.4^c^	9.0 ± 0.0^a^	12.7 ± 0.7^b^
18:0	4.2 ± 0.1^b^	4.1 ± 0.1^b^	2.3 ± 0.0^a^	2.5 ± 0.0^a^
Σ SFA	32.8 ± 0.6^c^	34.0 ± 2.8^c^	15.0 ± 0.0^a^	23.3 ± 2.1^b^
16:1 ω7	5.7 ± 0.1^b^	7.3 ± 0.2^c^	2.5 ± 0.0^a^	5.9 ± 0.4^b^
18:1 ω9	5.6 ± 0.1^a^	6.9 ± 0.1^a^	38.8 ± 0.2^c^	30.5 ± 0.8^b^
20:1 ω9	0.6 ± 0.0^a^	1.7 ± 0.0^b^	2.9 ± 0.0^c^	2.7 ± 0.2^c^
22:1 ω11 + ω9	4.7 ± 0.1^d^	3.7 ± 0.2^c^	0.4 ± 0.0^a^	1.8 ± 0.1^b^
Σ MUFA	22.6 ± 0.0^a^	23.5 ± 0.3^a^	47.9 ± 0.2^c^	41.3 ± 0.8^b^
18:2 ω6	1.4 ± 0.0^a^	1.0 ± 0.0^a^	14.3 ± 0.0^c^	9.2 ± 0.2^b^
20:4 ω6	1.6 ± 0.0^c^	1.0 ± 0.2^b^	0.4 ± 0.0^a^	0.5 ± 0.0^a^
18:3 ω3	0.7 ± 0.0^a^	0.6 ± 0.0^a^	5.5 ± 0.0^c^	3.3 ± 0.1^b^
18:4 ω3	1.9 ± 0.0^c^	2.1 ± 0.1^d^	0.9 ± 0.0^a^	1.5 ± 0.1^b^
20:4 ω3	0.7 ± 0.0^a^	0.6 ± 0.1^a^	1.1 ± 0.0^b^	0.7 ± 0.1^a^
20:5 ω3	10.8 ± 0.2^b^	15.3 ± 2.0^c^	2.9 ± 0.0^a^	7.1 ± 0.5^ab^
22:5 ω3	2.1 ± 0.0^b^	1.4 ± 0.1^a^	1.3 ± 0.0^a^	1.2 ± 0.1^a^
22:6 ω3	17.8 ± 0.3^c^	12.0 ± 0.0^b^	5.7 ± 0.0^a^	6.0 ± 0.4^a^
Σ PUFA	41.4 ± 0.1^b^	39.5 ± 2.6^ab^	34.8 ± 0.0^a^	34.5 ± 1.3^a^
Σ ω3	35.6 ± 0.1^b^	34.4 ± 2.3^b^	18.2 ± 0.0^a^	21.7 ± 1.1^a^
Σ ω6	4.6 ± 0.0^b^	3.0 ± 0.2^a^	16.1 ± 0.0^d^	10.7 ± 0.3^c^
Σ ω3/Σ ω6	7.7 ± 0.1^c^	11.6 ± 0.1^d^	1.1 ± 0.0^a^	2.0 ± 0.0^b^

Values are presented as average ± *SD*. Different lowercase letters within a row correspond to statistical differences (*p* < 0.05).

#### 3.1.2 Lipid fraction

The distribution of the main lipid classes in the selected fish samples (A, B, and C) and supplement prior to digestion is displayed in Tables 2 and 3. Samples of fish A and B showed some hydrolysis of their TAG class, thereby explaining their lower share of TAGs in fat and the detection of free fatty acids (FFAs). Fish C exhibited no hydrolysis and a very high level of TAGs in the total lipid fraction, 90.4 ± 0.2%. Likewise, cod liver oil supplement did not display any hydrolysis. Regarding the other lipid classes, no difference was found between fish samples. The share of diacylglycerol (DAG) and cholesterol (CHL) (mostly CHL) was always under 10%, and phospholipid (PL) class represented always a similar small share in the 4%–5% range. No monoacylglycerols (MAGs) were detected.

**Table 2:** Distribution of main lipid classes (%) in the three fish samples with different fat contents before (initial) and after digestion (bioaccessible)

Initial versus Bioaccessible	Lipid classes					
	FFA	TAG	CHL + DAG	MAG	PL	Other
Fish A (5.4% lipid)						
Initial	10.9 ± 0.8^aA^	75.4 ± 1.7^aA^	8.3 ± 2.7^aA^	0.0 ± 0.0^aA^	5.3 ± 0.2^aA^	0.0 ± 0.0^aA^
Bioaccessible	67.8 ± 4.5^bA^	0.0 ± 0.0^bA^	18.9 ± 0.4^aA^	10.7 ± 4.1^bA^	2.6 ± 0.1^aA^	0.0 ± 0.0^aA^
Fish B (10.2% lipid)						
Initial	8.8 ± 1.5^aA^	79.3 ± 2.4^aA^	6.9 ± 1.0^aA^	0.0 ± 0.0^aA^	5.0 ± 2.9^aA^	0.0 ± 0.0^aA^
Bioaccessible	76.5 ± 0.6^bB^	0.0 ± 0.0^bA^	8.8 ± 1.1^aB^	12.1 ± 1.9^bA^	2.7 ± 0.3^aA^	0.0 ± 0.0^aA^
Fish C (16.6% lipid)						
Initial	0.0 ± 0.0^aB^	90.4 ± 0.2^aB^	5.4 ± 1.2^aA^	0.0 ± 0.0^aA^	4.2 ± 1.0^aA^	0.0 ± 0.0^aA^
Bioaccessible	64.5 ± 7.8^bA^	0.0 ± 0.0^bA^	13.0 ± 6.1^aAB^	21.1 ± 1.3^bB^	1.3 ± 0.4^aA^	0.0 ± 0.0^aA^

Values are presented as average ± *SD*. For each fish, different lowercase letters within a column correspond to statistical differences between the initial and the bioaccessible samples (*p* < 0.05). For the initial or bioaccessible samples, considered separately, different uppercase letters within a column correspond to statistical differences between the fish samples (*p* < 0.05).

**Table 3:** Distribution of main lipid classes (%) in the cod liver oil supplement before (initial) and after digestion (bioaccessible), taking into account the different tested amounts in the digestion model

Initial versus Bioaccessible	Amount of cod liver oil (mg)	Lipid Classes					
		FFA	TAG	CHL + DAG	MAG	PL	Other
Initial	—	0.0 ± 0.0	74.5 ± 1.9	25.5 ± 1.1	0.0 ± 0.0	0.0 ± 0.0	0.0 ± 0.0
Bioaccessible	82	73.3 ± 0.8^bA^	0.0 ± 0.0^aA^	0.0 ± 0.0^aB^	13.1 ± 1.9^bA^	0.0 ± 0.0^aB^	13.6 ± 1.1^aB^
	154	70.4 ± 1.2^bB^	0.0 ± 0.0^aA^	0.0 ± 0.0^aB^	15.4 ± 0.9^bA^	0.0 ± 0.0^aB^	14.2 ± 0.2^aB^
	313	46.6 ± 5.1^aB^	22.6 ± 1.4^bB^	4.8 ± 0.1^bB^	12.3 ± 2.2^abB^	0.1 ± 0.0^aB^	13.6 ± 4.2^aB^
	604	40.8 ± 2.7^a^	26.5 ± 2.6^b^	7.7 ± 0.6^c^	10.2 ± 0.7^ab^	0.2 ± 0.0^a^	14.9 ± 1.3^a^
	1,027	36.8 ± 4.1^a^	31.9 ± 0.1^c^	11.2 ± 1.1^d^	7.0 ± 0.5^a^	0.6 ± 0.1^b^	12.7 ± 3.7^a^

Values are presented as average ± *SD*. Different lowercase letters within a column correspond to statistical differences between in vitro digestion trials using different amounts of cod liver oil (*p* < 0.05). Different uppercase letters in the three‐first rows correspond to differences to the matched fish samples after digestion (bioaccessible) in Table 2 (*p* < 0.05): 82 mg—Fish A; 154 mg—Fish B; 313 mg—Fish C.

As fat level increases from fish A (5.4% ± 0.3% w/w) to fish B (10.2% ± 0.1% w/w) and C (16.6% ± 0.2% w/w), it is expected an increase in the share of storage lipids, mainly found as TAG (Stubhaug, Tocher, Bell, Dick, & Torstensen, 2005). In fact, the TAGs were highest in the fattest fish sample. However, the expected reduction of the PL share in total fat was not observed. The PL range is similar to that found in other studies involving fish with more than 5% w/w fat, such as gilthead seabream (Costa et al., 2016), and lower than the range of 8%–10% w/w observed in leaner fish, with <5% w/w fat, such as sole (Afonso et al., 2017). The TAG levels and the absence of MAGs were also reported in previous studies with other fish species, such as gilthead seabream (Costa et al., 2016) and salmon (Costa et al., 2015). In this latter case, a very fat fish (19.9% ± 0.8% w/w), just as in fish C, TAGs also exceeded 90% of total lipid and no FFA was detected.

#### 3.1.3 Fatty acid profile

The FA profiles of the fish samples (A, B, and C) as well as of the cod liver oil supplement are shown in Table 1. There were important differences between the four samples. Whereas the PUFAs were the main group of FAs in the FA profiles of fish samples A and B, the monounsaturated FAs (MUFAs) were the most abundant FAs in fish sample C and in the supplement. On the other hand, the saturated FAs (SFAs) were lower in these samples, being the lowest share of SFA displayed by fish C. The differences in the SFA contents were mainly due to the variations in the palmitic acid (16:0) concentration. Nonetheless, there was also a contrast between samples A/B and sample C in other SFAs, such as myristic acid (14:0) and stearic acid (18:0). Regarding levels of MUFAs, the main contributor for the observed variations was oleic acid (18:1 ω9) whose concentration was highest in fish sample C, reaching 38.8 ± 0.2%. The nearest oleic acid content was that of the oil supplement, 30.5 ± 0.8%, given the very low levels of oleic acid in samples A and B, below 10%. The reverse was observed for palmitoleic (16:1 ω7) and docosenoic acids (22:1 ω11 + ω9), being the lowest levels found in fish C. Concerning PUFAs, ω3 PUFAs’ concentration variation between samples was opposite to the ω6 PUFAs’ variation. Samples A and B formed again a group distinct of sample C and supplement. Indeed, while ω3 PUFA content decreased from 34%–36% (A and B) to 18%–22% (C and supplement), the level of ω6 PUFA augmented from 3%–5% (A and B) to 11%–16% (C and supplement). Of course, this entailed a steep reduction in the ω3/ω6 ratio from 8–12 (A and B) to 1–2 (C and supplement). Specifically, the most relevant ω3 PUFAs were EPA and DHA, which were much less abundant in sample C and the oil supplement. Linolenic acid (18:3 ω3) content was almost as high as DHA value in fish C. It is also noteworthy that the highest EPA level was determined in fish B and the highest DHA level in fish A (the leanest of all fish samples). In keeping with the total ω6 PUFA content, linoleic acid (18:2 ω6) content also increased from approximately 1% (A and B) to 9%–14% (C and supplement).

The abundance of MUFA in fish C is similar to the same phenomenon observed in other farmed fish species, such as salmon (Costa et al., 2015). The low ω3/ω6 ratio measured in fish C is not much different of the ω3/ω6 ratio in raw farmed salmon, 1.6 (Costa et al., 2015). Furthermore, the proportions of the main FAs (oleic, palmitic, linoleic, and DHA) in fish C are similar to the values found in farmed salmon (Costa et al., 2015; Nanton et al., 2007). Any differences may be ascribed to the particular feed used in rearing farmed fish and not to any other factor. On the other hand, values found for fish samples A and B are similar to FA profiles of other wild fish species reported in the literature (Bandarra et al., 1997, 2001). Regarding the oil supplement, its FA composition differed in some aspects of other cod liver oils (Lei et al., 2016), especially in the presence of a substantial share of linoleic acid that was the cause of a low ω3/ω6 ratio. Nonetheless, its richness in oleic acid and ω3 PUFA was comparable to the values reported in the literature (Lei et al., 2016).

### 3.2 FA Bioaccessibility

#### 3.2.1 Degree of hydrolysis

The level of TAG hydrolysis in the bioaccessible fractions of the fish and supplement samples is presented in Tables 2 and 3. The degree of hydrolysis as measured by the disappearance of TAGs was complete in all fish samples, thus indicating a fully carried out digestive process. This entailed the formation of substantial amounts of MAGs and FFAs. PL hydrolysis did not show significant differences. Moreover, as a result of conversion of TAG to DAG and MAG, FFAs had a steep increase with respect to the initial (before digestion) samples. The comparison between the bioaccessible fractions of each fish sample showed that in fish C a higher share of MAG remained after digestion and in fish B a low share of DAG was coupled with a high percentage of FFA after digestion. Hence, results suggest a more complete hydrolysis of the ester bonds in the lipids of fish B.

Regarding the supplement, total disappearance of the TAG band was only observed for the lower amounts of oil fed to the in vitro digestion system, 82 and 154 mg of oil. Moreover, for the highest amount of supplement, 1,027 mg, an even higher amount of TAG was detected. Conversely, the share of FFAs in the bioaccessible fractions clearly declined from 70%–73% to 37%–47% with oil amounts of 313 mg or higher. There was also an increase in the DAG percentage from this oil amount upwards. The reduction of MAGs together with a further increase in DAGs in the case of the highest amount of oil is also meaningful. Even in the case of PLs, there was a slight increase in the percentage when the largest load of oil was fed to the model system.

Therefore, while the raw fish samples’ digestion was successful, there were problems concerning digestion of 313 mg and larger quantities of cod liver oil supplement. This leads to the comparison between raw fish and oil samples. It must be considered that 1.5 g (quantity used in the in vitro model) of fish A, B, and C corresponds to approximately 81, 153, and 249 mg of fat, respectively. Therefore, samples A, B, and C are matched by 82, 154, and, less perfectly, 313 mg of cod liver oil supplement. The most meaningful differences are found between fish C and 313 mg of oil. However, it must be reminded that 313 mg is 26% higher than the fat quantity in fish C.

Taken together, these results do not support any loss of efficiency by the lipases as a result of more complex matrices (as is the case in raw fish samples). This means that fish proteins and other constituents do not interfere in lipolysis. This absence of interference of the fish chemical composition is a first major insight that was aimed at by this study of raw fish *vs* oil. Such interference could be due to constituent–lipid chemical interactions or modified rheological properties of the digestate containing other constituents (Amyoony, Lin, & Wright, 2017; Lamothe, Rémillard, Tremblay, & Britten, 2017). Specifically, it has been claimed that specific proteins may affect bioaccessibility of other nutrients (Moser, Chegeni, Jones, Liceaga, & Ferruzzi, 2014). However, it has also been reported that lipases are more interfacially active than other proteins and may prevail at the interface even in the presence of large amounts of other proteins (Reis et al., 2008).

Results also seem to indicate that the used in vitro digestion model operates successfully up to quite high fat levels in fish. These may be, at least, 16% w/w or even 20% w/w, if salmon data from a previous study using the same model are taken into account (Costa et al., 2015). On the other hand, any sample with 300 mg or more fat may require changes to the model, for instance, demanding higher levels of lipase.

#### 3.2.2 Bioaccessible fatty acid profile

The fatty acid profiles of the bioaccessible fractions of the three fish samples (A, B, and C) and of the cod liver oil supplement (fed to the in vitro model at 82, 154, 313, 604, and 1,027 mg of oil) are shown in Tables 4 and 5, respectively.

**Table 4:** Fatty acid profile (% of total FAs) of the bioaccessible fraction of the three fish samples

	Fish A (5.4% lipid)	Fish B (10.2% lipid)	Fish C (16.6% lipid)
14:0	8.9 ± 0.1^cA^	8.1 ± 0.0^bA^	1.8 ± 0.0^aA^
16:0	21.1 ± 3.3^bA^	17.4 ± 1.0^bA^	7.7 ± 0.0^aA^
18:0	3.8 ± 1.9^aA^	1.6 ± 0.7^aA^	2.1 ± 0.0^aA^
Σ SFA	37.8 ± 6.0^bA^	29.4 ± 1.9^bA^	13.2 ± 0.0^aA^
16:1 ω7	8.2 ± 0.6^bB^	11.1 ± 0.2^cB^	2.3 ± 0.0^aA^
18:1 ω9	6.2 ± 0.3^aB^	8.3 ± 0.0^bB^	43.4 ± 0.1^cB^
20:1 ω9	3.4 ± 0.1^cB^	2.1 ± 0.0^bB^	0.1 ± 0.1^aB^
22:1 ω11 + ω9	6.4 ± 0.4^cB^	4.3 ± 0.0^bB^	2.2 ± 0.0^aB^
Σ MUFA	29.9 ± 1.7^aB^	30.6 ± 0.1^aB^	55.2 ± 0.1^bB^
18:2 ω6	0.0 ± 0.0^aB^	0.1 ± 0.0^aB^	14.1 ± 0.0^bB^
20:4 ω6	0.3 ± 0.4^aB^	0.6 ± 0.1^aA^	0.0 ± 0.0^aA^
18:3 ω3	0.7 ± 0.1^aA^	0.7 ± 0.0^aA^	5.3 ± 0.0^bB^
18:4 ω3	2.0 ± 0.3^bA^	2.5 ± 0.1^bA^	0.8 ± 0.0^aA^
20:4 ω3	0.6 ± 0.1^aA^	0.6 ± 0.0^aA^	1.0 ± 0.0^bA^
20:5 ω3	9.6 ± 1.5^bA^	15.7 ± 0.6^cA^	2.5 ± 0.1^aA^
22:5 ω3	1.8 ± 0.3^aA^	1.4 ± 0.1^aA^	1.2 ± 0.0^aA^
22:6 ω3	11.8 ± 1.1^bB^	10.5 ± 0.5^bA^	3.8 ± 0.0^aA^
Σ PUFA	32.4 ± 4.3^aB^	39.7 ± 1.8^aA^	31.7 ± 0.1^aA^
Σ ω3	28.2 ± 3.5^bB^	34.2 ± 1.5^bA^	15.4 ± 0.1^aA^
Σ ω6	1.8 ± 0.5^aB^	1.7 ± 0.2^aB^	15.7 ± 0.0^bA^
Σ ω3/Σ ω6	16.3 ± 3.0^bB^	20.2 ± 1.3^bB^	1.0 ± 0.0^aA^

Values are presented as average ± *SD*. Different lowercase letters within a row correspond to statistical differences between fish samples (*p* < 0.05). Different uppercase letters correspond to differences to the same fish samples before digestion in Table 1 (*p* < 0.05).

**Table 5:** Fatty acid profile (% of total FAs) of the bioaccessible fractions of the cod liver oil supplement simulating different fat intake levels

	82 mg oil	154 mg oil	313 mg oil	604 mg oil	1,027 mg oil
14:0	3.1 ± 0.4^aB^	4.9 ± 0.1^bA^	4.0 ± 0.3^abA^	4.9 ± 0.4^bA^	5.0 ± 0.7^bA^
16:0	11.2 ± 1.1^aA^	11.2 ± 0.3^aA^	9.3 ± 0.4^aA^	11.5 ± 0.3^aA^	11.5 ± 0.9^aA^
18:0	3.7 ± 0.2^bA^	2.5 ± 0.1^aA^	2.1 ± 0.3^aA^	2.4 ± 0.0^aA^	2.6 ± 0.0^aA^
Σ SFA	19.2 ± 1.7^aA^	19.7 ± 0.4^aA^	16.5 ± 0.4^aB^	20.0 ± 0.6^aA^	20.4 ± 1.7^aA^
16:1 ω7	4.7 ± 0.4^aA^	6.0 ± 0.0^abA^	5.5 ± 0.5^abA^	6.0 ± 0.1^bA^	5.6 ± 0.4^abA^
18:1 ω9	34.0 ± 0.1^abB^	34.0 ± 0.6^abB^	34.5 ± 0.4^abB^	35.0 ± 0.3^bB^	33.3 ± 0.1^aB^
20:1 ω9	3.0 ± 0.1^aA^	2.8 ± 0.0^aA^	3.2 ± 0.3^aA^	2.9 ± 0.0^aA^	3.2 ± 0.2^aA^
22:1 ω11 + ω9	2.4 ± 0.3^aA^	1.9 ± 0.1^aA^	2.3 ± 0.3^aA^	2.0 ± 0.1^aA^	2.3 ± 0.1^aA^
Σ MUFA	44.5 ± 1.0^aB^	45.0 ± 0.5^aB^	45.8 ± 0.6^aB^	46.2 ± 0.2^aB^	44.7 ± 0.1^aB^
18:2 ω6	12.0 ± 0.5^cB^	11.2 ± 0.1^bcB^	10.7 ± 0.4^abB^	10.1 ± 0.2^abA^	9.6 ± 0.1^aA^
20:4 ω6	1.7 ± 0.2^cB^	1.1 ± 0.0^bB^	0.9 ± 0.0^abB^	0.7 ± 0.0^aA^	0.6 ± 0.0^aA^
18:3 ω3	2.7 ± 0.2^aB^	3.0 ± 0.0^aA^	3.2 ± 0.2^aA^	3.1 ± 0.1^aA^	3.1 ± 0.0^aA^
18:4 ω3	1.1 ± 0.1^aB^	1.2 ± 0.0^aA^	1.3 ± 0.2^aA^	1.3 ± 0.1^aA^	1.2 ± 0.1^aA^
20:4 ω3	0.6 ± 0.0^aA^	1.1 ± 0.0^aA^	0.7 ± 0.0^aA^	0.6 ± 0.0^aA^	0.7 ± 0.0^aA^
20:5 ω3	5.5 ± 0.6^aB^	5.7 ± 0.3^aA^	6.8 ± 0.3^aA^	5.8 ± 0.1^aA^	6.3 ± 0.5^aA^
22:5 ω3	1.2 ± 0.1^aA^	1.2 ± 0.1^aA^	1.4 ± 0.1^aA^	1.0 ± 0.1^aA^	1.3 ± 0.1^aA^
22:6 ω3	6.2 ± 0.3^aA^	5.6 ± 0.4^aA^	6.8 ± 0.3^aA^	5.5 ± 0.1^aA^	6.5 ± 0.6^aA^
Σ PUFA	35.1 ± 2.3^aA^	34.1 ± 0.9^aA^	36.4 ± 0.9^aA^	32.4 ± 0.4^aA^	33.5 ± 1.4^aA^
Σ ω3	18.7 ± 1.6^aA^	18.9 ± 0.9^aA^	21.8 ± 0.4^aA^	18.8 ± 0.2^aA^	20.5 ± 1.3^aA^
Σ ω6	14.8 ± 0.7^cB^	13.3 ± 0.1^bcB^	12.7 ± 0.3^abB^	11.8 ± 0.2^abA^	11.3 ± 0.2^aA^
Σ ω3/Σ ω6	1.3 ± 0.0^aB^	1.4 ± 0.1^abB^	1.7 ± 0.0^cdB^	1.6 ± 0.0^bcB^	1.8 ± 0.1^dA^

Values are presented as average ± *SD*. Different lowercase letters within a row correspond to statistical differences between different amounts of digested cod liver oil (*p* < 0.05). Different uppercase letters correspond to differences to the cod liver oil before digestion in Table 1 (*p* < 0.05).

Concerning fish samples, there was a balanced distribution between the total SFA, MUFA, and PUFA in fish A and B, while total MUFAs were clearly the most abundant group and total SFAs the least abundant group in fish C. Though palmitic acid content was higher in fish A and B than in fish C, it was the most abundant SFA in all samples. Whereas the palmitoleic acid was the most abundant MUFA in A and B, oleic acid was the most abundant FA across all FA groups in fish C, reaching 43.4% ± 0.1%. With respect to ω6 PUFA, their levels were below 2% in fish A and B—linoleic acid was undetectable or almost absent—being fish C the only sample with a large share of ω6 PUFA, exceeding 15%. On the other hand, ω3 PUFA content was higher in fish A and B (28.2%–34.2% of total FAs) than in fish C (15.4%)—EPA and DHA had levels of 10% and larger each. As a consequence, the bioaccessible ω3/ω6 ratio was very high in fish A and B, reaching 16 and 20 in A and B, respectively, and low in C (ω3/ω6 ratio of 1).

These bioaccessible FA profiles had similarities with the initial (before digestion) FA profiles (compare Tables 4 and 1). Namely, total SFA levels were similar. However, there were differences. The total MUFA and oleic acid contents were higher in the bioaccessible fraction. The total ω6 PUFA and linoleic acid levels in the bioaccessible FA profiles of fish A and B were lower than in the samples prior to digestion. No differences were found in ω3 PUFA in fish B and C. Owing to digestion, the ω3/ω6 ratio did not change in fish C, but it was increased, almost doubling, in fish A and B.

The bioaccessible FA profiles of the oil trials were all very similar. The only clear trend was observed in the percentages of ω6 PUFA and linoleic acid, which declined with increasing amount of supplement. In comparison with the supplement FA profile before digestion (Table 1), total MUFA and oleic acid contents were higher in all five bioaccessible fractions. For supplement amounts up to 313 mg, there was also an increase in ω6 PUFA and linoleic acid in the bioaccessible fraction, thus causing a deterioration of the ω3/ω6 ratio.

On the basis of raw fish and oil samples’ results, it can be stated that total MUFA and oleic acid are enriched in the bioaccessible fraction. A similar study centered on sole did not show any enrichment of these FAs as a result of digestion (Afonso et al., 2017). Likewise, this sole study did not find any systematic increase or decrease in ω6 PUFA and linoleic acid concentrations in the bioaccessible fractions. Taking into account supplements’ trials, ω6 PUFA and linoleic acid seem to be more concentrated in relative terms in the bioaccessible fractions than before digestion up to an amount of fat corresponding to approximately a 20% w/w fat fish (300 mg in 1.5 g). As lipolysis becomes incomplete at higher fat amounts, the relative advantage of these FAs seems to vanish. Hence, ω6 PUFA and linoleic acid may well be more abundant in the bioaccessible fractions, thus suggesting a bioaccessibility above the average FA bioaccessibility. Variables such as lipolysis kinetics (Giang et al., 2016), solubilization in bile salt mixed micelles (Freeman, 1969), and emulsion properties (Zhang, Zhang, Zhang, Decker, & McClements, 2015) may contribute to determine, for instance, a lower or higher bioaccessibility of linoleic acid than that of other FAs.

#### 3.2.3 Calculation of the bioaccessibility factors of lipid and fatty acids

The calculated values of the lipid and FA bioaccessibility factors in percentage for the tested fish and supplement samples showed differences between FAs and samples (Table 6).

**Table 6:** Lipid and fatty acid bioaccessibility (%) of the three fish samples and cod liver oil corresponding to different fat intake levels

	Fish samples bioaccessibility (%)			Cod liver oil samples bioaccessibility (%)				
	Fish A (5.4% lipid)	Fish B (10.2% lipid)	Fish C (16.6% lipid)	82 mg oil	154 mg oil	313 mg oil	604 mg oil	1,027 mg oil
Lipid	78 ± 2^AB^	75 ± 14^A^	98 ± 2^B^	85 ± 3^AB^	88 ± 5^AB^	93 ± 1^AB^	92 ± 0^AB^	93 ± 4^AB^
14:0	90 ± 5^deAB^	95 ± 2^cB^	80 ± 1^bAB^	51 ± 7^aA^	72 ± 2^abAB^	59 ± 8^abcAB^	68 ± 6^aAB^	71 ± 16^aAB^
16:0	94 ± 15^eB^	67 ± 9^bcAB^	82 ± 0^bAB^	56 ± 12^abA^	63 ± 6^aAB^	57 ± 1^abA^	71 ± 3^aAB^	75 ± 13^aAB^
Σ SFA	88 ± 14^deA^	67 ± 10^bcA^	84 ± 0^bA^	55 ± 10^abA^	62 ± 5^aA^	56 ± 2^aA^	69 ± 4^aA^	73 ± 13^aA^
18:1 ω9	85 ± 2^deA^	93 ± 1^cAB^	100 ± 0^cB^	93 ± 3^cAB^	91 ± 6^cA^	95 ± 5^dAB^	93 ± 1^dAB^	91 ± 7^aA^
Σ MUFA	85 ± 5^deA^	100 ± 2^cAB^	100 ± 0^cB^	93 ± 5^cAB^	91 ± 6^cA^	94 ± 5^dAB^	91 ± 1^cdA^	91 ± 8^aA^
18:2 ω6	0 ± 0^aA^	4 ± 3^aA^	94 ± 0^cC^	75 ± 0^abcB^	81 ± 6^bcBC^	89 ± 10^dBC^	84 ± 0^bcdBC^	84 ± 7^aBC^
18:3 ω3	81 ± 9^cdeA^	86 ± 1^cA^	91 ± 0^cA^	79 ± 5^bcA^	80 ± 4^bcA^	85 ± 12^bcdA^	76 ± 1^abA^	77 ± 6^aA^
20:5 ω3	68 ± 8^cdeA^	80 ± 13^bcA^	81 ± 3^bA^	76 ± 7^bcA^	72 ± 0^abA^	84 ± 9^abcdA^	68 ± 0^aA^	75 ± 1^aA^
22:6 ω3	51 ± 5^bcA^	67 ± 3^bcB^	64 ± 1^aB^	98 ± 3^cE^	82 ± 2^bcCD^	98 ± 2^dE^	75 ± 2^abBC^	91 ± 0^aDE^
Σ PUFA	60 ± 7^bcdA^	78 ± 8^bcAB^	87 ± 0^bB^	79 ± 4^bcAB^	78 ± 2^abcAB^	88 ± 8^cdB^	75 ± 0^abAB^	81 ± 4^aAB^
Σ ω3	61 ± 7^bcdA^	77 ± 8^bcAB^	81 ± 0^bB^	82 ± 5^cB^	77 ± 0^abcAB^	88 ± 7 ^dB^	71 ± 0^aAB^	80 ± 2^aAB^
Σ ω6	30 ± 8^abA^	44 ± 8^bA^	93 ± 0^cB^	75 ± 0^abcB^	79 ± 6^abcB^	89 ± 9 ^dB^	83 ± 0^bcB^	84 ± 6^aB^

Values are presented as average ± *SD*. For each sample, different lowercase letters within a column correspond to statistical differences (*p* < 0.05) between fatty acids. For lipid content and each fatty acid, different uppercase letters within a row correspond to statistical differences between samples (*p* < 0.05).

With the exception of the highest amount of oil supplement, 1,027 mg, the FAs’ bioaccessibility percentages were variable. In the case of oil amounts between 82 and 604 mg, the bioaccessibility of SFA was always lower than that of MUFA. This agrees with the relative enrichment of MUFA in the bioaccessible fraction of these samples. The bioaccessibility percentage of PUFA was mostly between those values of SFA and MUFA. In fact, the bioaccessibility of each PUFA varied widely. Linoleic acid displayed very low bioaccessibility levels in fish A and B. DHA bioaccessibility was sometimes lower than the bioaccessibilities of oleic acid and MUFA (for instance, in fish A and C as well for 604 mg oil), but never higher. Furthermore, bioaccessibility of EPA was sometimes lower than that of oleic acid and MUFA (in fish C and 154 and 604 mg oil), but never higher. Hence, there were some trends, namely, bioaccessibility of EPA and DHA was typically lower than that of oleic acid and MUFA.

Regarding the comparison between raw fish and oil samples, overall lipid bioaccessibility was lower in fish B than in fish C, being all the other samples intermediate. At a more detailed level, while there was no difference in the SFA bioaccessibility between samples, MUFA and PUFA bioaccessibility percentages of fish A were lower than those of fish C. This was also found for ω3 PUFA bioaccessibility, 61% ± 7% (A) vs. 81% ± 0% (C), and ω6 PUFA bioaccessibility, 30% ± 8% (A) vs. 93% ± 0% (C). For particular FAs, the same opposition between fish A and fish C was found in oleic acid, linoleic acid, and DHA. Concerning the oil supplements, no difference was detected among them except for DHA bioaccessibility. However, there was no clear trend for this parameter.

The relationship between SFA, MUFA, and PUFA bioaccessibility was different of what was reported for salmon (Costa et al., 2015), sole (Afonso et al., 2017), and gilthead seabream (Costa et al., 2016). Specifically, for sole fed a diet low in linoleic acid and gilthead seabream, the bioaccessibility of PUFA was lower than that of SFA and MUFA. Nevertheless, in the consulted literature (Afonso et al., 2017; Costa et al., 2016), the highest bioaccessibility values have been mostly ascribed to MUFA group, just as in the current study. The gilthead seabream study (Costa et al., 2016) also reported an EPA and DHA bioaccessibility lower than MUFA bioaccessibility. Very low linoleic acid bioaccessibility values were determined in sole fed a diet low in linoleic acid (Afonso et al., 2017) and gilthead seabream (Costa et al., 2016). On the other hand, salmon (Costa et al., 2015) and sole fed a diet rich in linoleic acid (Afonso et al., 2017) presented bioaccessibility percentages for linoleic acid similar to MUFA bioaccessibility. This parallels the findings of the current fish and oil study. Therefore, linoleic acid bioaccessibility seems to be consistently dependent on its initial amount.

Moreover, results only support a reduction of bioaccessibility for higher levels of unsaturation, given the variation between SFA and MUFA bioaccessibility—a first double bond does not seem to be deleterious to FA bioaccessibility. Concerning this effect of the unsaturation level, it is possible that lipases are less able to hydrolyze more unsaturated FAs (Giang et al., 2016), thus hindering their bioaccessibility. This can be supported by recent work on PPL, which has found that PPL preferentially hydrolyzes FAs whose first double bond from the ester linkage is farther from the ester (Akanbi, Sinclair, & Barrow, 2014). Precisely, less unsaturated FAs, such as MUFA, have in average a double bond farther apart from the ester linkage. Nonetheless, less lipolysis may be insufficient to explain lower bioaccessibility. Accordingly, bioaccessibility differences between FAs may be due to the chemical affinity of their forms (for instance, oleic acid, monoolein, glyceryl dioleate, glyceryl trioleate, etc.) to the undigested protein in the nonbioaccessible fraction in comparison with the substances in the bioaccessible fraction. The higher polarity of PUFAs, such as EPA and DHA, with respect to MUFA may explain a lower bioaccessibility of these highly unsaturated FAs as observed in the current study. The chemical affinity issues may be related to emulsion difficulties. Indeed, it has also been reported that the uptake of EPA and DHA is especially increased by pre‐emulsification (Garaiova et al., 2007) and it is favored by utilization of gelled emulsions instead of traditional oil supplements (Haug et al., 2011).

The attained results show that the fat level of the samples did not affect percentage of bioaccessibility. Though lipolysis was reduced with increasing amounts of oil supplement, no detrimental effect was detected in bioaccessibility of total lipids or some of the most abundant FAs. Accordingly, lipolysis and bioaccessibility may not correlate. This deserves further investigation, given the existence of other studies that detected some correlation between lipolysis and lipophilic substance (carotenoid) bioaccessibility (Amyoony et al., 2017).

Regarding the raw fish *vs* oil comparison for highlighting the effects of fish chemical composition on FA bioaccessibility, the interference of protein and other components on lipid and FA bioaccessibility was almost absent in the fattest fish sample (C), given the similarity of the bioaccessibility values of fish C sample (249 mg of fat) and the oil supplement amount of 313 mg, except for DHA. In the latter case, there was a lower bioaccessibility level that may be ascribed to the presence of proteins and other nonlipid constituents in fish C. However, these effects were apparently more significant in fish A and B samples. This was observed in ω6 PUFA, linoleic acid, and DHA bioaccessibility parameters (comparison of fish A containing 81 mg of fat with 82 mg of oil supplement and of fish B containing 153 mg of fat with 154 mg of oil supplement). Therefore, it seems that a reduction of lipid and FA bioaccessibility by interaction of the lipid components with proteins and other components becomes more important when the relative weight of the lipid fraction is smaller with respect to that of the protein fraction (Table 1). The nature of the interaction may involve viscosity and physical properties of the digestate (Amyoony et al., 2017) or chemical mechanisms and adsorption phenomena (Rein et al., 2013).

Finally, it should be noted that this study did not aim to draw conclusions about the advantage of consuming either oil supplements or fish, but focused on the effects of fat level and the interference of other nutrients on lipolysis and bioaccessibility. For this reason, raw fish was used, thereby excluding thermal treatment effects as a factor. Cooking would necessarily affect FA bioaccessibility and, probably, reduce it (Costa et al., 2015), but a comparison between cooked fish and oil supplements for a critical dietary appraisal, particularly with respect to ω3 PUFA and DHA, must be the focus of future study.

## 4 CONCLUSIONS

It was possible to make valuable comparisons on the basis of the fish and oil supplement samples used in this study. Firstly, it must be mentioned that digestion of the fish and oil (up to 154 mg) samples as measured by TAG disappearance was complete. Lipolysis was impaired by high amounts of oil (313 mg and higher). There was no loss of efficiency by the lipases as a result of more complex matrices—containing nonlipid components. Bioaccessible FA profiles had similarities with the initial (before digestion) FA profiles. However, total MUFA and oleic acid contents were higher in the bioaccessible fraction. The bioaccessibility of EPA and DHA was typically lower than that of oleic acid and total MUFA. Results support a reduction of bioaccessibility for higher levels of unsaturation in fish samples. This may be due to a lower level of lipolysis or to chemical phenomena specific of the highly unsaturated FAs. Moreover, results show that the fat level of the samples did not affect bioaccessibility. Accordingly, lipolysis and bioaccessibility may be difficult to correlate. On the other hand, protein and other components may have interfered in lipid bioaccessibility and it was found that the reduction of bioaccessibility was stronger when the relative weight of the lipid fraction was smaller with respect to that of the protein fraction.

## AUTHOR CONTRIBUTIONS

RG and SM performed experimental work concerning the bioaccessibility trials and related analyses, CC interpreted the results and wrote the manuscript, CA and NB designed the study, and NB coordinated the whole study.

## CONFLICT OF INTEREST

The authors declare that they do not have any conflict of interest.

## ETHICAL STATEMENT

Ethical Review: This study did not involve any human or animal testing. Moreover, human and animal testing was unnecessary in this study.
